# Where Are the Sore Losers? Competitive Authoritarianism, Incumbent Defeat, and Electoral Trust in Zambia’s 2021 Election

**DOI:** 10.1093/poq/nfae030

**Published:** 2024-07-16

**Authors:** Nicholas Kerr, Matthias Krönke, Michael Wahman

**Affiliations:** Assistant Professor, Department of Political Science, University of Florida, Gainesville, FL, US; Researcher, Institute for Democracy, Citizenship and Public Policy in Africa, University of Cape Town, Cape Town, South Africa; Associate Professor, Department of Political Science, Michigan State University, East Lansing, MI, US

## Abstract

How do electoral turnovers shape citizen perceptions of election quality in competitive authoritarian regimes? We argue that electoral outcomes are crucial for determining perceptions of electoral quality. While detailed evaluation of electoral trust is complex in competitive autocracies with institutional uncertainty and polarized electoral environments, turnovers send strong and unequivocal signals about election quality. Previous literature has noted a strong partisan divide in electoral trust in competitive authoritarian regimes, but turnovers can boost trust among both incumbent and opposition supporters. We test this argument in the case of Zambia’s 2021 election, a case where a ruling party lost despite electoral manipulation and strong control over the Election Management Body (EMB). Empirically, we leverage the first-ever panel survey carried out during Zambian elections. Comparing trust in elections before and after the election, we find that perceived election quality increased after the 2021 electoral turnover among both losers and winners. We find that trust in elections increased the most among winning opposition supporters. Moreover, despite the outgoing president’s attempt to portray the election as fraudulent, losing ruling-party supporters also increased their trust in elections after the turnover. The study has important implications for the literature on democratic consolidation and institutional trust.

## Introduction

For most citizens in competitive authoritarian regimes, evaluating the quality of elections and the performance of electoral institutions is difficult (Kerr 2013, 2018). Electoral processes are logistically complex and regulated by technical legal instruments and international norms. Electoral manipulation is by its very nature concealed, and losing parties have incentives to undermine electoral credibility to obscure their poor electoral performance. Moreover, compared to supposedly consolidated democracies (e.g., Gibson et al. 2003), voters in competitive autocracies will find it much harder to rely on independent government institutions, a vibrant civil society, or a free press to assess the quality of elections.

The informationally complex environment of competitive authoritarian elections invites voters to evaluate elections based on their partisan biases. With conflicting and ambiguous evaluations of election quality, they are likely to prioritize information from partisan sources and confirm their prior beliefs about institutional fairness or bias (Robertson 2017). It is hardly surprising that research from across competitive authoritarian contexts has shown great partisan divides in evaluations of electoral quality where winners trust elections and electoral institutions significantly more than election losers (Moehler 2009; Cantú and García-Ponce 2015; Flesken and Hartl 2018).

How may incumbent defeat in competitive autocracies change popular perceptions about electoral fairness? Will turnovers change trust in elections along expected partisan lines, or will they enhance trust in elections among both government and opposition supporters? In this paper, we argue that turnovers can break familiar patterns of partisan motivated reasoning among voters. When institutions are generally perceived to systematically favor the incumbent, voters are likely to perceive turnovers as proof of high election quality.

In more democratic countries such as Brazil and the United States, populist incumbents have recently questioned the integrity of elections after suffering defeat (Berlinski et al. 2021; Enders et al. 2021; Filho et al. 2022). For example, devoted Republican partisan supporters have reacted to accusations of electoral manipulation of the presidential elections by lowering their trust (Stewart 2022) and blaming their loss on the proestablishment bias of government institutions characteristic of the “deep state.” In competitive authoritarian regimes, however, incumbent regimes before losing have usually defended the embattled records of government institutions in response to persistent opposition scrutiny and criticism. While competitive autocratic leaders may still dispute the quality of lost elections, discrediting electoral institutions among supporters will be harder when these institutions have historically been viewed as treating the government party favorably.

We utilize a new election panel survey from Zambia’s 2021 election. The panel includes more than 1,300 Zambian voters interviewed shortly before and shortly after the election. Election panel surveys remain extremely rare in the developing world among competitive authoritarian regimes.^1^ Moreover, since competitive authoritarian regimes favor incumbents, turnovers are not commonly captured in such panels. Zambia is a useful case for the purpose of this study. Despite the electoral turnover, the Electoral Commission of Zambia (ECZ) showed obvious progovernment bias in the run-up to the election. Moreover, the election resulted in a landslide victory for the opposition, but the losing incumbent candidate (President Edgar Lungu) claimed the election was fraudulent. Given this context, Zambia makes for a difficult test of the hypothesis that turnovers increase electoral trust across partisan affiliations.

Competitive authoritarian regimes have been characterized as multiparty regimes in the gray zone between autocracy and democracy. Levitsky and Way (2010, p. 12) argue that “whereas full authoritarian regimes are characterized by the absence of competition (and, hence uncertainty) and democracy is characterized by fair competition, competitive authoritarianism is marked by competition that is real but unfair.” Turnovers are rare, but not impossible, in competitive authoritarian regimes. While there are certainly examples of more repressive competitive autocracies than Zambia, the country has consistently been classified as an electoral autocracy by the Varieties of Democracy project since 2013. The fact that Zambia is less autocratic than many other competitive autocracies (such as Angola, Uganda, and Zimbabwe) means that any effect that we find in Zambia would most likely be even larger in other competitive autocracies where turnovers are less probable.^2^

Our regression analyses show that Zambians, regardless of partisan affiliation, increased their trust in elections after the August 2021 election. Voters supportive of the winning opposition party increased their trust in elections significantly more than voters of the losing incumbent party. However, trust in elections increased significantly, even among losing ruling-party supporters. Remarkably, trust in elections increases even among the strongest government-party supporters.

This study has important implications for our understanding of perceptions of electoral integrity and democratic consolidation. The results suggest that perceptions of election quality are not simply a consequence of motivated reasoning. On the contrary, even election losers are willing to upgrade their perceptions of election quality. While informational environments are often complex and politicized, voters use crude signals about procedural strength to assess election quality. Our results also suggest that overall assessments of election quality heavily depend on evaluations of the counting and aggregation of votes. Our findings show that even voters who were heavily critical of the pre-electoral environment are ready to reevaluate their overall assessment of elections if election results suggest a fairly accurate counting of the vote. These findings have implications for our understanding of how different forms of electoral manipulation may affect popular perceptions of electoral integrity.

## Trust in Competitive Authoritarian Elections

According to the influential definitions set forward by Levitsky and Way (2010), competitive authoritarian regimes are defined by their unfair electoral competition. Incumbents in competitive authoritarian regimes use the powers of the state to manipulate every aspect of elections (Schedler 2002). They legislate to manipulate electoral rules in their favor and use state resources to manipulate voters’ preferences. They sometimes even manipulate the tallying of the vote (Birch 2011). Trust in electoral systems characterized by such systemic manipulation may appear puzzling. Still, voters in competitive autocracies as diverse as Russia, Venezuela, and Zimbabwe have shown reasonably high levels of trust in elections, at least in some partisan groups (Rose and Mishler 2009; Maldonado and Seligson 2014; Krönke 2018).

Trust in fraudulent elections may result from a general appreciation of electoral outcomes or, more generally, difficulty in evaluating election integrity on an election-by-election basis. The level of manipulation in competitive authoritarian regimes is not constant across time. Indeed, institutional uncertainty is the lifeblood of competitive authoritarianism (Schedler 2013). Elections in this context create stability and regime legitimacy, but only if they display a minimum of credibility (Magaloni 2006). The continuous participation of opposition parties is contingent on a sense that elections create a somewhat credible path to power (Beaulieu 2014).

To enhance credibility in the electoral process, authoritarian regimes set up formally autonomous institutions to organize elections and adjudicate electoral disputes (Chernykh and Svolik 2015). While incumbent regimes often interfere with the functioning of such institutions, they have also been known to acquire increased autonomy and professional ethos over time (Lindberg 2006). The process of democratization in competitive authoritarian regimes is often gradual and not necessarily unidirectional (Teorell 2010). Thus, voters and political actors can rarely be certain about the extent of manipulation that will be used in any given election (Seeberg 2014; Rundlett and Svolik 2016). The frequent mismatch between how institutions work on paper and their de facto performance creates uncertainty and variability in institutional trust. Further, this variability creates a focus on short-term performance, which is very different from supposedly consolidated democracies where elections are institutionalized and enjoy deeper legitimacy.

Institutional uncertainty leads to varying prospects for free and fair elections, but the informational election environment in competitive authoritarian regimes is generally poor and politicized. The freedom of the press is commonly curtailed, and state-owned media outlets are heavily biased in favor of the government (VonDoepp and Young 2013; Kerr and Lührmann 2017). While international or local election observers can potentially provide information about elections, such organizations have their own political agendas and are often discredited or misquoted by domestic political actors (Kelley 2009; Hyde 2011; Bush and Prather 2018). Domestic election observers are commonly politicized and made out to be pro-opposition by incumbent regimes (McDonald and Molony 2023). Moreover, supposedly independent institutions such as courts and election commissions are biased and beholden to incumbent regimes, making them unreliable sources for evaluating the integrity of elections (Kerr and Wahman 2021).

## Election Turnover and Trust in Elections

When institutional uncertainty is high and information environments are poor and politicized, how much will voters rely on electoral outcomes to make crude judgments on the quality of elections? Elections in competitive authoritarian regimes are designed to protect the interests of the incumbent and diffuse the threats from a disempowered opposition. Nevertheless, while electoral manipulation mitigates electoral pressure, it does not guarantee regime survival (Wahman 2014; Bleck and van de Walle 2019). When regime popularity dwindles and a strong opposition successfully challenges the incumbent, competitive authoritarian elections occasionally result in electoral turnovers (Bunce and Wolchik 2011).

Elections represent a long cycle, where manipulation can occur at varying stages (Birch 2011; Norris 2013). Modern-day manipulation often occurs long before election day. Electoral integrity may be undermined in the registration of voters, in the delimitation of electoral boundaries, by the arbitrary enforcement of the electoral code, or through the use of rogue strategies such as vote buying or election violence, to mention but a few examples (Cheeseman and Klaas 2018). Nevertheless, for voters living in information-poor competitive authoritarian regimes, election results are likely to play a central role in evaluating the quality of the electoral process. While messages from parties, civil society, government institutions, the media, and international observers are often ambiguous and contradictory, there is less ambiguity about election results.

In competitive authoritarian regimes, voters will assume that manipulation mostly favors the institutionally and resource-advantaged incumbent regime. If government parties win elections, the outcome will likely reaffirm the notion that elections are biased in favor of the ruling party. Rare cases of opposition victory can, however, challenge many preconceived notions about elections. If elections end in incumbent defeat, it may be hard for voters to imagine that the will of the people was significantly distorted. That is, the information provided by election results will likely trump more ambiguous information about electoral fairness in earlier parts of the election process. Voters do not need in-depth information about electoral laws or detailed and reliable reporting of compliance with electoral codes of conduct to interpret the meaning of a turnover. Electoral turnovers may thus serve to drastically change perceptions of voters, even those who prior to elections expected the process and outcome to be highly biased in favor of the incumbent. We, hence, formulate H1:

*H1: Turnovers increase overall trust in elections.*

It seems reasonable to expect that electoral turnovers increase trust in elections among opposition supporters (e.g., the winners). After all, they will have few complaints about an election outcome that promotes their interests (Anderson et al. 2005). The harder test, however, is whether turnovers will also enhance trust among those supporting the losing incumbent.

Multiple studies in authoritarian and democratic settings alike have found that partisan winners are more likely than partisan losers to embrace elections as free and fair (e.g., Moehler 2009; Moehler and Lindberg 2009; Edelson et al. 2017; Levy 2021). *Motivated reasoning theory* suggests that voters tend to focus on procedural deficiencies that benefit partisan opponents and downplay the procedural deficiencies that may have benefited their favored party. In social psychology, it is commonly argued that individuals’ personal predispositions influence their evaluations when information is complex or ambiguous (Kunda 1990). When elections are lost, voters are likely to see the electoral defeat as proof of procedural deficits. In contrast, winners are more likely to believe that the election was a true reflection of the popular will. Partisan differences in evaluations are particularly likely to diverge when losers do not acknowledge defeat or claim fraud (Hernández-Huerta and Cantú 2021; Esaiasson et al. 2022).

Earlier research has shown significant motivated reasoning in relation to incumbent reelection, the most common outcome in competitive authoritarian regimes (Robertson 2017). In relation to incumbent reelection, incumbent supporters will rely on official government communication to evaluate election quality but will not be persuaded by allegations made by opposition sources (Robertson 2017; Mochtak et al. 2021). Opposition supporters, on the other hand, have generally low baseline trust in government institutions and will be suspicious of the information put out by such official sources. They will instead rely on the assessment made by opposition parties or affiliated information channels.

The motivated reasoning thesis would predict that turnovers should have a divergent effect on evaluating election quality. While opposition supporters should improve their perceptions of election quality after their party prevailed, incumbent-party supporters should have lower perceptions of election quality after the surprising electoral defeat. However, recent public opinion research has argued that the motivated reasoning thesis may have been overstated. According to Coppock (2022), when different groups hold different opinions at baseline, opinions often move in similar directions and to a similar extent (in parallel) if people are presented with *credible* information promoting updates of prior beliefs.

Considering competitive authoritarian elections, the unexpected event of an electoral turnover may serve as credible information that will offset typical motivated reasoning. Government-party voters are likely to have higher baseline trust in the integrity of elections. If elections lead to opposition victory, incumbent losers should be less likely to consider this a product of election manipulation. While incumbent losers may still try to undermine the credibility of elections after defeat, they will be hard pressed to discredit the institutional context they have consistently lauded as credible and impartial during periods where they have strongly benefited from institutional bias. With both government and opposition supporters aware of institutional biases, both are likely to see an incumbent defeat as an unexpected sign of electoral integrity. As a consequence, both government and opposition supporters alike are likely to increase their trust in elections after turnovers:

*H2: Turnovers increase trust in elections among both incumbent and opposition supporters.*

## Case Selection and Study Context

Zambia’s 2021 election is a useful case to study the effect of turnovers on public trust in elections in competitive authoritarian regimes. First, several important elements of the election should have made it harder to improve the perceptions of electoral integrity among government-party supporters. Second, Zambia illustrates the difficulty for losing incumbent regimes to undermine the trust in elections organized by heavily biased institutions.

**Figure 1.**
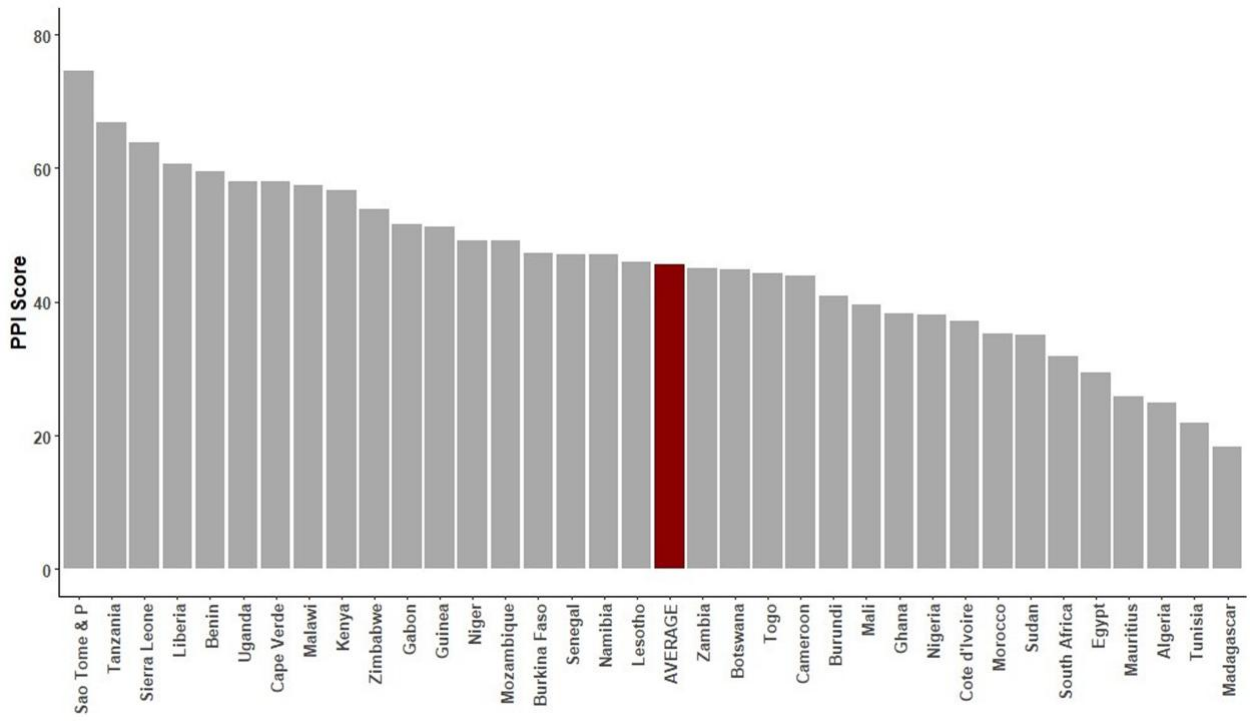
Free and fair elections. Percent of respondents who say the most recent election was completely free and fair, or free and fair with minor problems | 2019/2021 | 34 countries. *Source*: Afrobarometer 2022.

According to V-Dem data, Zambia has been consistently categorized as “electoral authoritarian” since 2013 (Coppedge et al. 2022). Regarding trust in elections, Zambia only scores close to the African average according to cross-national Afrobarometer data (see figure 1). Furthermore, trust in elections is highly dependent on partisanship, and lower trust among opposition supporters may be explained by significant pro-incumbent institutional bias.

The 2021 Zambian election occurred in the context of serious democratic backsliding and a deep economic crisis. Since taking office after a by-election in 2015 and being reelected in a regular election in 2016, President Lungu presided over a regime that politicized, censored, and undermined the independence of courts, civil society, and the free press (Hinfelaar et al. 2022a; Hinfelaar et al. 2022b).

The 2016 election was highly controversial and affected by violence, institutional bias, and restrictions in opposition campaigns (Goldring and Wahman 2016; Wahman 2023). What is more, the opposition claimed that the election had been rigged on behalf of the government. While the opposition filed a petition to the Constitutional Court, the judges on the Lungu-appointed court had dismissed the petition on technical grounds (Ndulo 2016; Sishuwa 2016). To this day, the opposition United Party for National Development (UPND) maintains that the election was stolen. After the election, polarization increased further, not least after 2017 when the opposition leader, Hakainde Hichilema, was imprisoned for 100 days on trumped-up treason charges.

Going into the 2021 election, the institutional bias from courts and the Electoral Commission of Zambia (ECZ) was clear. The Constitutional Court had again shown its bias by ruling that Lungu had the right to run for a third term in office, despite the constitution’s two-term limit. The ECZ had a long tradition of pro-incumbent bias, stemming from the executive’s significant powers to appoint commissioners and interfere with the commission’s operations (Helle 2016; Kaaba and Haang’andu 2020). The Chairperson of the ECZ at the time of the 2021 election, Esau Chulu, had been personally appointed by President Lungu in 2015. After the controversial election of 2016, Chulu became a divisive figure, lauded by the PF but heavily criticized by the UPND (Goldring and Wahman 2016).^3^

In 2021, the ECZ bias was particularly evident in the handling of the crucial registration process, which resulted in a much larger increase in registered voters in government-party strongholds than in opposition areas (Kaaba and Haang’andu 2021; Resnick 2022). It was also noticeable in the ECZ’s arbitrary enforcement of COVID-19 campaign restrictions, where the commission limited the opposition’s ability to campaign without putting the same restrictions on the PF (Kaaba and Haang’andu 2021).

The election ended in a resounding defeat of President Lungu and his PF and a decisive victory for Hichilema and his UPND. Hichilema won the 2021 election with great margins (59 percent vs. 39 percent) and a larger vote total than any other candidate in Zambian electoral history (Siachiwena 2022a). The turnout was also extraordinary at 70 percent, the highest turnout in any Zambian election since 2006. The high turnout ultimately provided much-needed legitimacy to the election. Generally, Zambians’ willingness to come out and vote has been interpreted as a deep desire for change and a strong rejection of the PF regime (Siachiwena 2022b).

While Zambia did experience a turnover, it is important to note that turnover itself is not proof that the election was free and fair and without institutional bias (Beardsworth et al. 2022). Before the election, influential public intellectual Sishuwa Sishuwa famously concluded, “In a fair election, Lungu can’t win. In an unfair one, he can’t lose” (Sishuwa 2021). However, one might instead argue that Lungu lost *despite* an unfair election. This is supported by the EU election mission’s preliminary statement (EUEOM 2021, p. 1), which concluded that the campaign period was “marred by unequal campaign restrictions, restrictions on freedoms of assembly and movement, and abuse of incumbency.” Similarly, Beardsworth et al. (2022, p. 522) conclude that the electoral turnover was particularly surprising “because it came amidst deepening autocratization and the efforts of an incumbent who did everything possible to secure reelection including manipulating state resources, controlling the media, judiciary, and security forces, and enacting various antimedia laws to intimidate the opposition and suppress voting.”

The defeat shows some of the limitations incumbents face in trying to discredit election results in competitive autocracies. Lungu could not credibly maintain that a system that had predictably acted in his favor—and that he had consistently lauded for its professionalism—had denied him his rightful victory. Still, he tried. As results were trickling in and the inevitability of a PF defeat became clear, President Lungu announced that the election had “not been free and fair” and demanded that it be nullified (Beardsworth et al. 2021). To buy time, the government shut down the internet, and the ECZ drastically slowed down the release of election results. Nevertheless, the government was unable to maintain the hardline approach. After strong pressure from other African heads of state and international monitors, and with little sign of popular support for the struggling incumbent, Lungu decided to concede defeat and not to file a petition with the Constitutional Court (Kaaba et al. 2022).

The Zambian case allows us to measure the effect of the turnover on public trust in elections among opposition and incumbent supporters. It is particularly interesting to observe government-party supporters, given the government’s attempt to paint the election as flawed. Zambia is also a conservative test, as the turnover is not the first in the country’s history. According to data from the Afrobarometer survey, trust in elections increased among both government and opposition supporters after the last Zambian turnover in 2011 (see Supplementary Material figure A1),^4^ but it is possible that the effects of turnovers decline when voters become more accustomed to such events. If we find an effect in Zambia, we are also likely to find similar or stronger effects in other competitive autocracies with fewer experiences of turnovers.

## Data and Measurement

**Figure 2.**
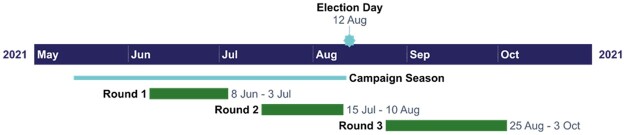
Timing of survey rounds of Zambian Election Panel Survey. The solid line represents the official campaign period. The green bars represent the ZEPS fieldwork periods, while the blue star signifies the election day.

The data used in this paper are sourced from an original three-round panel survey, the Zambian Election Panel Survey (Lust et al. 2021), and contain approximately 1,300 respondents drawn from 74 districts during and after the 2021 Zambian campaigns and elections (figure 2). The three rounds of the panel were implemented by phone between 8 June and 3 October 2021: Round 1 (early campaign: June–July); Round 2 (late campaign: July–August); Round 3 (post-election: August–October) (see figure 2). To ensure that we capture the effect of the electoral turnover, rather than other confounding effects (e.g., events during the first half of the campaign period), we use data from the two rounds closest to the election (rounds 2 and 3). It is important to note that Round 3 was administered after President Hichilema took office. It is possible that respondents saw his final swearing-in as further proof that the election was legitimate and certified by all important players.

Panel surveys outside established Western democracies remain extremely rare. To the best of our knowledge, no previous panel data from competitive authoritarian regimes have been able to capture an electoral turnover to determine how such events correspond with updated beliefs on election quality in a panel of voters.^5^ While earlier research has used cross-sectional Afrobarometer data to study the correlation between turnovers and perceptions of election quality in Africa (Moehler and Lindberg 2009), this research has been limited by long time intervals elapsing between survey rounds.

**Figure 3.**
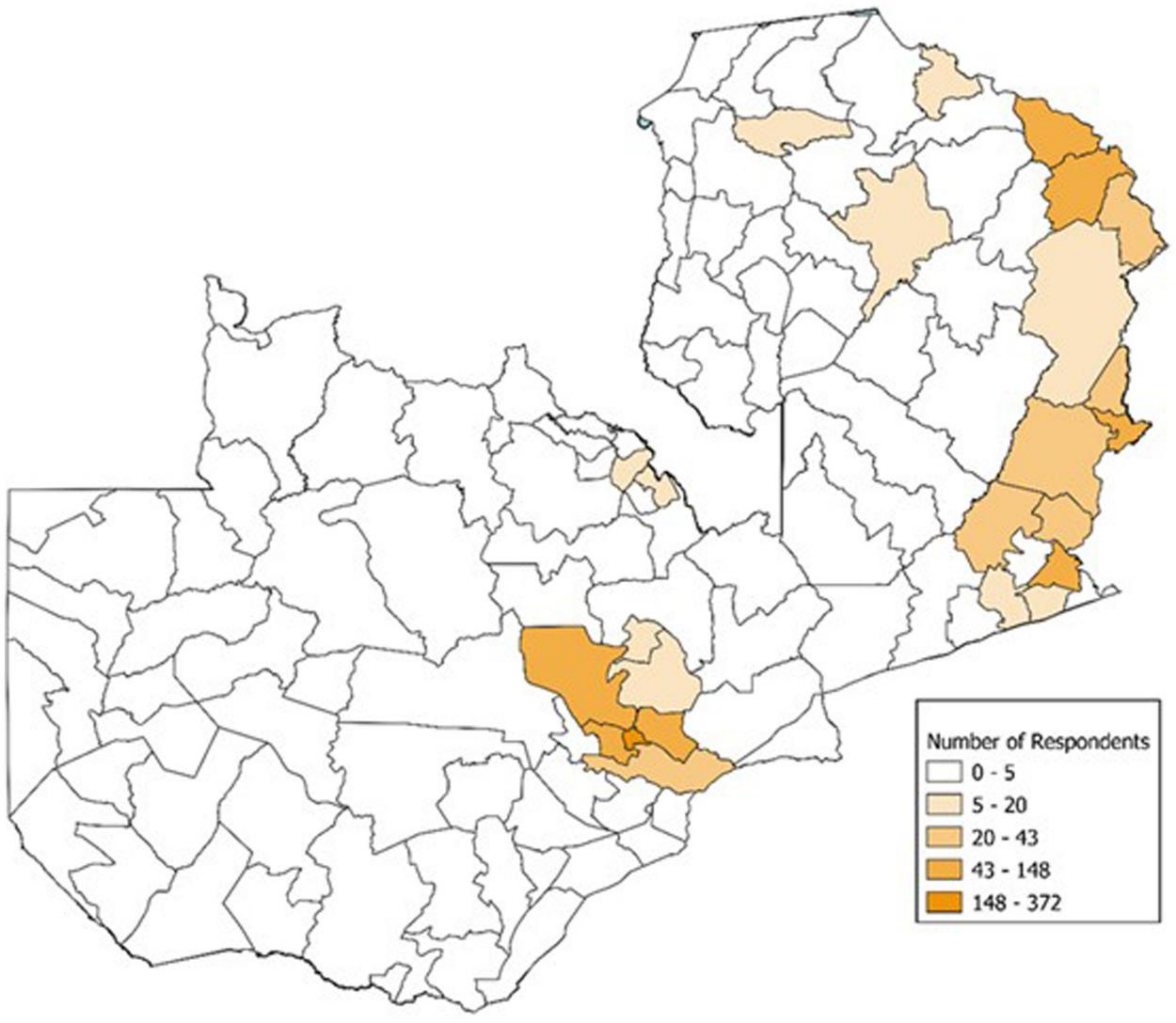
Distribution of respondents per district in Round 3. *Source*: Lust et al. (2021).

The sample used for our panel survey is not nationally representative and cannot be used to make inferences about general levels of election trust across Zambia (see figure 3). It can, however, register the change over time in attitudes among sampled individuals. The sampling frame was built on previous face-to-face surveys fielded since 2019 (Lust et al. 2021). The original survey sample was obtained via a stratified multistage probability proportional to size sampling scheme. The strata included two regions: (1) a 50 km radius of Lusaka, and (2) a 100 km region from the Zambia-Malawi border. For additional technical information about the survey sample as well as to what extent it differs from the country as a whole, please see Supplementary Material section B.

While not nationally representative, the survey sample is skewed toward government-party strongholds. This sample design makes for a conservative test of our hypothesis. We expect that informational bias will be particularly strong in government-party strongholds, and that government-party voters would be particularly likely to evaluate election quality based on motivated reasoning if they update their beliefs in a pro-government informational environment (Horowitz and Long 2016; Letsa 2019).

### Dependent Variable

To measure perceptions of election quality, we rely on the question asked in Rounds 2 and 3: “On the whole, how free and fair do you expect that the August 2021 presidential election will be/was?” Response options are coded on a 0–3 scale (0 = Not free and fair; 1= Free and fair with major problems; 2 = Free and fair, but with minor problems; 3 = Completely free and fair).^6^  Table 1 indicates that between R2 and R3 the mean election quality perception increased from 2.02 to 2.59. Substantively, this represents a 28 percent increase in average trust in elections and provides preliminary support for H1 that turnover is associated with increased trust in elections among all Zambians.^7^ Descriptive support for the salience of turnover following the 2021 election is reinforced if we consider that mean election quality perceptions between R1 and R2 only increased marginally by 3 percent (see Supplementary Material table C3).

**Table 1.** Change in perceptions of election quality by vote choice | Round 2 to Round 3.

	n	Round 2	Round 3	R3-R2	Δ% R2-R3	*p*
Pooled sample	1.209	2.02	2.59	0.56	28	0.00
By vote choice						
UPND	336	1.68	2.56	0.88	52	0.00
PF	454	2.25	2.58	0.33	15	0.00
Abstain	410	2.07	2.63	0.56	27	0.00
Others	9	1.11	2.11	1.00	90	0.04

*Note:* Cells indicate the number of respondents who answered questions on perceptions of election quality in Rounds 2 and 3; *p* values indicate results of a Wilcoxon signed-ranked test.

We construct the change in election quality perceptions measure by subtracting our measure of election quality perceptions in R2 from our measure in R3 (R3-R2). The change in election quality perceptions variable ranges from -3 to 3, with positive values indicating that respondents’ election quality perceptions increased between rounds, negative values indicating that perceptions decreased, and 0 indicating that perceptions remained unchanged. Change in election quality has a mean of 0.56 across our sample. Across all respondents, 46 percent (N = 566) increased their perceptions of election quality, while 10 percent (N = 119) decreased, and 43 percent (N = 524) did not alter their assessments of election quality.

### Independent Variables: Partisanship

While we find that, on average, Zambians increased their trust in elections between R2 and R3 of the survey, we are particularly interested in partisan differences. To measure citizens’ partisan affiliation, we rely on a question that asks respondents about their intended vote choice for the presidential election during the final stages of the campaign period (Round 2).^8^ We utilize intended vote choice measured in R2 instead of reported vote choice in R3 to minimize voters’ tendency to alter their reported voting decisions based on the outcome of elections (i.e., post-treatment bias). Overall, 35 percent (N = 537) said they would vote for the incumbent PF candidate, 27 percent (N = 410) said they would vote for the main challenger, Hakainde Hichilema from the UPND, while only 1 percent (N = 18) indicated other candidates (Other Party), and 37 percent answered Abstain, Don’t know, or Refused (Abstain).^9^

Table 1 displays the mean changes in perceptions of election quality across intended vote choice, respectively. The results from the bivariate analysis provide preliminary support for H2. Electoral turnover increases perceptions of election quality across all three categories, including UPND (0.88) and PF supporters (0.33). Again, these increases are non-trivial. For the average UPND supporters, trust in elections increased by 52 percent, whereas the average PF supporters’ trust increased by 15 percent.

### Control Variables

Although the bivariate findings are instructive, they do not allow us to effectively rule out the possibility that other individual and contextual factors may influence short-term changes in Zambians’ attitudes toward the integrity of the 2021 election. Guided by previous research on electoral integrity, we account for how the procedural aspects of elections, violence and intimidation, as well as demographic factors, influence changes in Zambian electoral legitimacy attitudes.

Zambians engage with the electoral process at various points, when registering for the election in the months and weeks before the election, as well as on election day when waiting in line to vote. Previous studies have shown that citizen evaluations of these procedural aspects are associated with their electoral trust (Hall et al. 2009; Kerr 2013). To measure the impact of the ECZ’s capacity to administer the election efficiently, we asked respondents in Round 3 about how satisfied they were with several aspects of the ECZ’s management of election processes at their polling station, including (1) accuracy of new voter register; (2) privacy of voting booths; (3) competence of polling staff; (4) impartiality of polling staff; (5) length of time it took to vote; and (6) transparency of the counting and announcement of results. We construct the *ECZ procedural capacity index* by creating an additive index, which is further transformed to a 0–1 scale with an increasing level of perceived capacity.

Past research has also shown that citizens who fear becoming victims of political intimidation or violence are less likely to believe that elections are credible (Kerr 2013). To gauge the impact of *election violence fear*, we use responses to a question measured in R2: “During the upcoming election, how much do you personally fear becoming a victim of political intimidation or violence?” Response options are coded on a 0–3 scale (0 = Not at all to 3 = A lot).

To gauge whether respondents’ political knowledge affects their level of electoral trust, we ask whether citizens *know the name of their elected representative (MP)*. Finally, we include a range of control variables that tap into individuals’ socioeconomic status. Specifically, we account for *age, gender, education, place of residence* ((peri-)urban vs rural), and *income*.

## Regression Results

We start our analysis by estimating the influence of partisanship (vote choice) on short-term changes in citizens’ perceptions of election quality, while controlling for other election-related and demographic factors. Again, our main expectations are that Zambians will increase their perceptions of election quality following the turnover in power (H1), and that turnover should boost trust in elections among opposition and government supporters (H2). The results of an OLS regression model are shown in table 2. Models 1A–B display the results of the analyses on changes in perceptions of election quality. We begin with the main indicators of intended vote choice (Model 1A), then include all the control variables (Model 1B). To ensure the robustness of the main results, we conduct additional sensitivity analyses (see Supplementary Material section C). We account for the ordinal nature of our dependent variable (Supplementary Material table C4), and the clustering of our respondents in enumeration areas (Supplementary Material table C5), and the main results remain substantially unchanged. We also account for the effect of floor or ceiling effects, a common challenge to interpreting results from panel data, by including Round 2 measure of perceptions of election quality as a control variable in the main regression analyses (see Supplementary Material table C6).^10^ The results from our robustness tests provide even stronger support for H2. While respondents, on average, report increased perceptions of election quality between R2 and R3, respondents cannot be distinguished by their vote choice, compared to the main findings where change in perceptions of election quality between R2 and R3 is significantly higher for UPND supporters and abstainers, relative to PF supporters (see Supplementary Material figure C2).

**Table 2.** Regression analysis, change in perceptions of election quality.

	(1)	(2)	(3)	(4)
	Model_2a		Model_2a	
	Change in perceived electoral quality of presidential election R3-R2		Change in perceived electoral quality of presidential election R3-R2	
VARIABLES	coef	*p* val	coef	*p* val

Vote choice pres (R2) = 0, UPND	0.550	(0.000)	0.524	(0.000)
Vote choice pres (R2) = 2, Abstain	0.233	(0.001)	0.240	(0.002)
ECZ procedural capacity			0.541	(0.003)
Fear violence (R2)			0.126	(0.000)
Know MP (R1)			−0.169	(0.021)
Age (R1)			0.003	(0.301)
Female (R1)			−0.042	(0.541)
Education (R1)			−0.003	(0.908)
Urban/peri-urban resident (R1)			0.049	(0.493)
Income (low)			0.062	(0.135)
Constant	0.328	(0.000)	−0.480	(0.092)
Observations	1,200		1,015	
R-squared	0.041		0.117	

*Note:* OLS regression, standard errors in parentheses.

**Figure 4.**
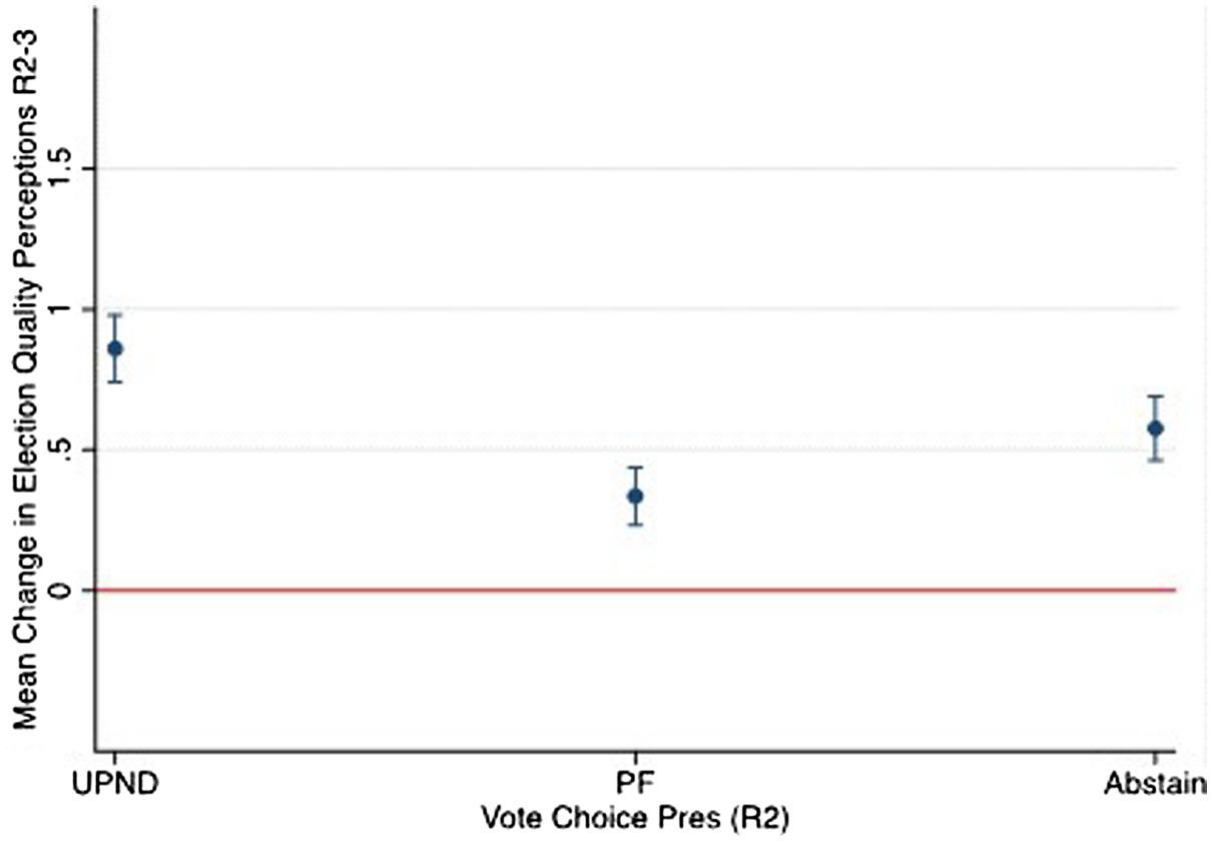
Predicted mean change in election quality perceptions by intended vote choice. The margins plot illustrates how the predicted mean change in election quality perceptions (R2) varies by respondents’ intended vote choice for the main political parties (based on table 2, Model 2B). Vertical bars indicate 95 percent confidence intervals.

For ease of interpretation, figure 4 displays the predicted mean changes in perceptions of election quality from R2 to R3 across citizens’ intended vote choice while keeping all controls at their mean response (based on Model 2B in table 2). We find support for H2, as all categories of citizens based on their voting intentions report increased perceptions of election quality. Recall that the *Change in election quality* variable runs from −3 to 3. Our model predicts a 0.86 (*p* = 0.00) increase among UPND voters and a 0.58 (*p* = 0.00) increase among those who abstain. More interesting is the fact that even among PF voters, whose presidential candidate lost the election, there is a predicted 0.34 (*p* = 0.00) increase in perceived election quality. In short, the evidence presented in figure 4 supports our hypothesis that electoral turnovers in competitive autocracies increase perceptions of electoral quality among both winners and losers.

**Figure 5.**
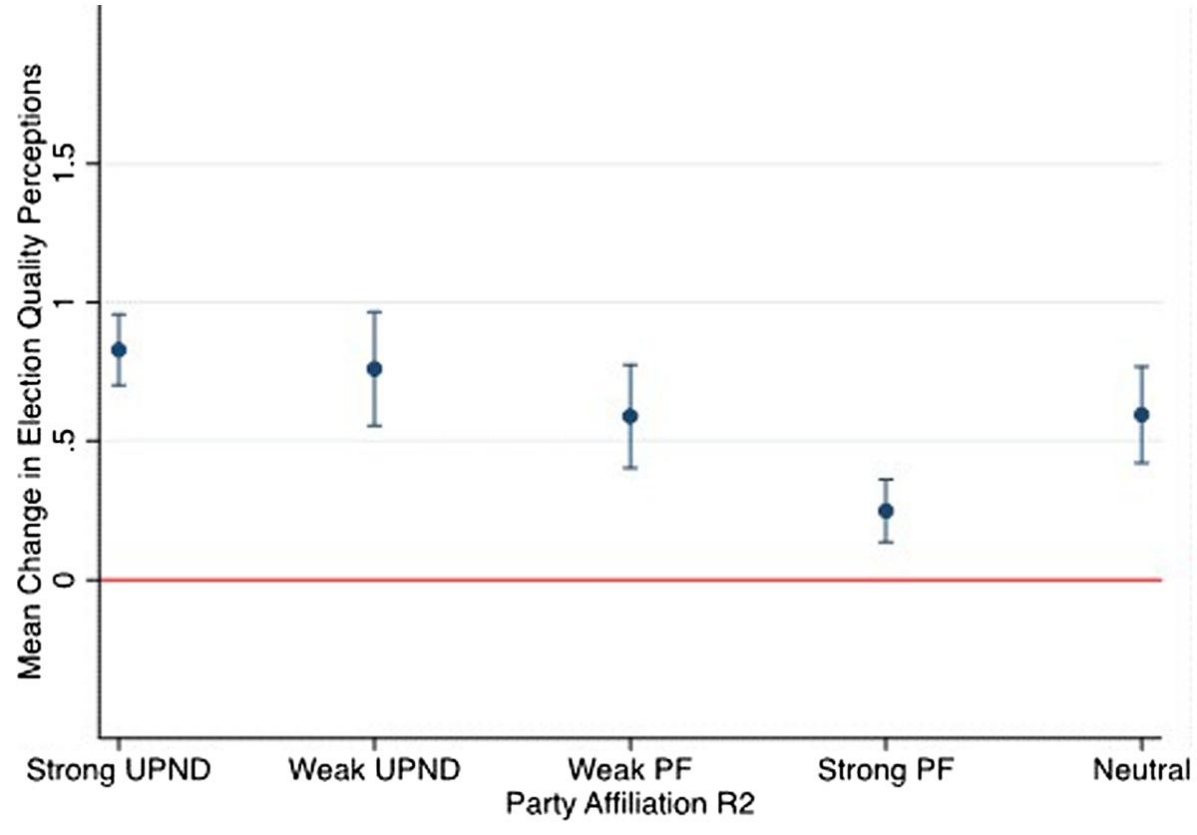
Predicted mean change in election quality perceptions by strength of partisanship. The margins plot illustrates how the predicted mean change in election quality perceptions (R2) varies by respondents’ strength of attachment to the main political parties (based on Supplementary Material table C8). Vertical bars indicate 95 percent confidence intervals. Strength of partisanship was measured in Round 2.

One potential limitation of the results concerning PF supporters is that the increase in election quality perceptions following the loss of the incumbent president is driven by PF voters with weak affiliation to the party. To explore this possibility, we rerun our main models using an alternative measure of party support that distinguishes respondents based on the strength of their attachment to the main political parties. We categorize respondents as strong or weak supporters of PF, strong or weak supporters of UPND, or neutrals (see Supplementary Material section C for more details on coding). Figure 5 displays the substantive results of our regression analyses using the alternative measures (see Supplementary Material table C8 for the regression results). Similar to the previous findings, all groups of respondents reported increased trust in elections, regardless of their strength of partisan attachment. In fact, we find that both strong and weak UPND supporters have virtually identical increases in perceptions of election quality following turnover (strong: 0.83; weak: 0.76). Meanwhile, the increase in trust is only marginally lower for weak PF supporters (0.6) relative to UPND supporters. However, even among the most ardent PF supporters, who we may expect to be critical of election quality following their party’s loss, trust in elections increases, albeit by a smaller amount (0.25).

Regarding the effect of the procedural aspects on electoral trust, our results are in line with previous findings. We show that citizens who had a more satisfactory experience at their local polling station are also more likely to report increased trust in elections. Respondents who were more worried about becoming a victim of political intimidation or violence prior to the election are more likely to have increased their trust in elections after election day. One plausible explanation is that the negative events citizens anticipated did not take place, or at least took place to a lower extent. Thus, their evaluations of elections increased after the election. Politically knowledgeable, those who could correctly identify their MP’s name, were more likely to downgrade their perceptions of the election after it took place (Bratton et al. 2005; Moehler 2009). Finally, respondent’s age, gender, level of education, income, as well as whether they live in a rural or urban area, had no statistically significant relationship with changes in the perceived integrity of elections. In addition to controlling for reported income, in our robustness checks, we also account for respondents’ perceived government economic performance measured in R2 (see Supplementary Material table C9). It is possible that even strong PF supporters who were dissatisfied with Lungu’s economic management may have increased their trust in elections, following his loss in the polls. However, even with the inclusion of this variable, the main substantive results remain unchanged.

## Conclusion

In competitive authoritarian regimes, elections and associated institutions do not enjoy deep legitimacy across partisan lines. Although voters have access to multiple sources of information to gauge the level of electoral integrity, they often provide contradictory assessments. In such ambiguous informational environments, motivated reasoning often takes hold, and partisan attachment highly affects the evaluation of elections.

 We used the Zambian presidential turnover in 2021 to argue that electoral turnovers in competitive authoritarian regimes are seismic events with the potential to mitigate motivated reasoning in relation to the evaluation of elections. Despite numerous factors surrounding the Zambian case that theoretically should promote a clearly partisan response to the electoral outcome, we find that Zambian citizens, regardless of partisan affiliation, enhanced their trust in elections after the historic electoral turnover. The empirical analysis of this study was enabled by a rare panel survey of Zambian voters.

The findings of this paper have several important implications. First, they speak to the importance of electoral outcomes for trust in elections and mitigate some of the strongest assumptions promoted by motivated reasoning theory. High levels of motivated reasoning are a significant challenge for the consolidation of new democracies. If the quality of elections is solely judged based on electoral outcomes, new democracies will struggle to establish basic institutional legitimacy. While our results here do not suggest the absence of motivated reasoning in the evaluation of election quality, they indicate that certain events have the potential to enhance electoral trust across partisan divides. The results of this paper particularly focus on short-term changes in perceptions of election quality, and more research is needed to understand the more long-term creation of electoral and institutional trust through electoral turnovers.

Second, the paper also makes important contributions to our understanding of how ordinary citizens judge electoral processes. Elections represent complex and long cycles, and electoral manipulation often happens long before the counting and tabulation of election results (Birch 2011; Norris 2013). Nevertheless, our results suggest that voters who may have been highly critical of the electoral conduct in the pre-electoral phase are ready to quickly update their evaluations of elections when outcomes suggest that the tabulation and aggregation of results were accurate.

Finally, our findings have important implications for theorizing the way in which different forms of electoral manipulation may affect public trust in elections. They support the understanding that more subtle forms of manipulation, those further removed from election day, may be less costly for authoritarian regimes wishing to manipulate the electoral outcome (Cheeseman and Klaas 2018). In extension, electoral commissions and other important electoral institutions have significant opportunities to hedge their bets in competitive authoritarian elections. In cases like Zambia, electoral commissions may play favorites with the incumbent regime, but withdraw such support when electoral defeat is inevitable. Public opinion highly determined by the conduct of the actual vote count reduces the costs of early cycle manipulation and creates significant opportunities for electoral institutions to engage in evolving tipping games. As a consequence, we may not expect that institutional bias will work in favor of the incumbent across the entire electoral cycle.

## Supplementary Material

nfae030_Supplementary_Data

## Data Availability

Replication data and documentation are available at: https://doi.org/10.7910/DVN/QNQCDP.

## Footnotes

1 Some previous examples include Conroy-Krutz and Kerr (2015) and Bartels et al. (2021).

2 For a comparison of Zambia compared to other electoral authoritarian regimes, please see Supplementary Material section A (table A1).

3 Chulu’s appointment with the ECZ was not renewed after Hichilema’s electoral victory (Zambia Daily Mail 2022).

4 While these data indicate that H1 and H2 may also be supported for the 2011 election, it is important to note that these are not panel data.

5
Bartels et al. (2021) employ a two-round panel to assess citizen evaluations of the Kenyan Supreme Court’s historic 2017 elections rulings, and Conroy-Krutz and Kerr (2015) study how campaigns and elections affect evaluations of democracy in transitional settings. Ugues and Vidal (2015) compare citizen evaluations of the electoral commission and court before and after the 2006 and 2012 Mexican elections. In the United States and Europe, these studies are somewhat more common (e.g., Esaiasson 2011; Levy 2021; Daniller and Mutz 2019).

6 “Don’t know” or refused to answer responses are treated as item-missing and excluded from the analysis. The wording of the question and the alternative responses are inspired from standard Afrobarometer question wording. Full details of all variables, including question wording and a description of all constructs and indices, can be found in Supplementary Material tables C1 and C2.

7 We report the marginal distributions in both bar graphs (Supplementary Material figure C1) and percentage tables (Supplementary Material table C3). These results corroborate the descriptive results on mean changes in perceptions of election quality between R2 and R3, but provide more detail about how respondents’ perceptions of election quality increased between rounds.

8 For this first stage of the analysis, we use the vote choice variable, as it is the best available option to capture the attitudes of PF and UPND supporters with that of nonpartisans (nonvoters). In the second part, we provide additional evidence using partisanship “thermometer” questions for the two main parties to show the differential effect of strength of partisanship. Both sets of results are in line with our theoretical expectations.

9 In subsequent analyses, we remove the 1 percent of respondents who indicated support for “other candidates.” The results of our main analyses remain unchanged if these respondents are included.

10 As an additional robustness check, we regress the Round 3 measure of perceptions of election quality on vote choice, the Round 2 measure of perceptions of election quality, and other covariates included in the main regression analysis (see Supplementary Material table C7 and figure C3). The results are identical to those in Supplementary Material table C6. We also run a model where we remove all UPND respondents who already have the highest level of trust in elections in R2. This is to account for possible ceiling effects (see Supplementary Material table C10).
