# Supplementary material for: Where Are the Sore Losers? Competitive Authoritarianism, Incumbent Defeat, and Electoral Trust in Zambia’s 2021 Election
Source: Public Opin Q. 2024 Jul 16;88(SI):608–31. doi: 10.1093/poq/nfae030 (PMC11300037; doi:10.1093/poq/nfae030)
Supplement: nfae030_Supplementary_Data [file nfae030_supplementary_data.pdf]

Supplementary Material to Accompany

Where are the Sore Losers?
Competitive Authoritarianism, Incumbent Defeat, and Electoral Trust in Zambia's 2021 Election

Nicholas Kerr
Department of Political Science
University of Florida

Matthias Krönke
Institute for Democracy, Citizenship and Public Policy in Africa
University of Cape Town, South Africa

Michael Wahman
Department of Political Science
Michigan State University
email: wahmanmi@msu.edu

Contents:

A. Study context and scope

B. Survey sample

C. Descriptive statistics and robustness checks

A: Study context and scope

Electoral authoritarian regimes across the world

To gage the scope of our results, we compiled a list of countries that, on average, were categorized as electoral autocracies by V-Dem between 2011 and 2021 (Table A1). That is, we include countries that scored between 0.5 and 1.4 on the three-point scale (0=closed autocracy; 1=electoral autocracy; 2=electoral democracy, 3=liberal democracy). Zambia (1.2) is among the more democratic and competitive regimes in this group of 61 countries (average=1.0), which means that the effect that we find in Zambia would most likely be even larger in other electoral autocracies where the information environment is poorer and turnovers are less probable.

Table A1. List of electoral authoritarian regimes | average 2011-2021

Country	Electoral authoritarian regime (0.5-1.4)	Country	Electoral authoritarian regime (0.5-1.4)
Afghanistan	0.9	Maldives	1.5
Algeria	1.0	Madagascar	0.8
Angola	1.0	Malaysia	1.0
Armenia	1.1	Maldives	1.5
Azerbaijan	1.0	Mali	1.5
Bangladesh	1.0	Mauritania	1.0
Belarus	1.0	Montenegro	1.2
Burma/Myanmar	0.9	Mozambique	1.0
Burundi	1.0	Nicaragua	1.0
Cambodia	1.0	North Macedonia	1.5
Cameroon	1.0	Pakistan	1.0
Central African Republic	1.0	Palestine/West Bank	1.0
Chad	0.9	Papua New Guinea	1.0
Comoros	1.0	Republic of the Congo	1.0
Dem. Republic of the Congo	1.0	Russia	1.0
Djibouti	1.0	Rwanda	1.0
Egypt	0.9	Serbia	1.3
Equatorial Guinea	1.0	Singapore	1.0
Ethiopia	1.0	Somaliland	1.0
Fiji	0.7	Sudan	0.7
Gabon	1.0	Tajikistan	1.0
Guinea	0.9	Tanzania	1.0
Haiti	1.0	The Gambia	1.2
Honduras	1.0	Togo	1.0
Iran	1.0	Turkey	1.2
Iraq	1.0	Turkmenistan	1.0
Ivory Coast	1.4	Uganda	1.0
Kazakhstan	1.0	Ukraine	1.2
Kenya	1.3	Venezuela	1.0
Kyrgyzstan	1.0	Zambia	1.2
Madagascar	0.8	Zanzibar	1.0
Malaysia	1.0	Zimbabwe	1.0

61 country average = 1.0

Source: Varieties of Democracy (Coppedge et al. 2022), v2x_regime.

Note: Electoral autocracy is measured as “de-jure multiparty elections for the chief executive and the legislature. but failing to achieve that elections are free and fair. or de-facto multiparty. or a minimum level of Dahl’s institutional prerequisites of polyarchy as measured by V-Dem’s Electoral Democracy Index (v2x_polyarchy).

Perceptions of free and fair elections over time in Zambia

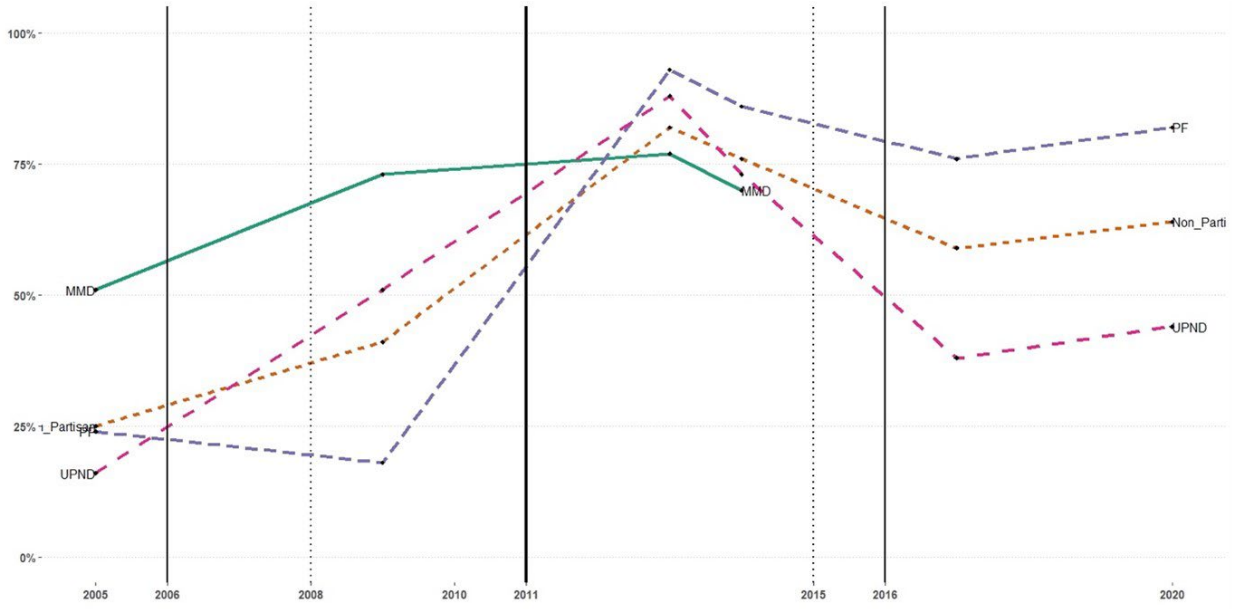

Figure A1. Free and fair elections in Zambia. Percent of respondents who say the most recent election was completely free and fair, or free and fair with minor problems | split by party identification | Zambia 2005 – 2020. Source: Afrobarometer 2022. Note: Purple dashed line = PF; Pink dashed lines = UPND; Orange dashed-dotted line = non-partisan; Green dolid line = MMD; Black vertical lines represent elections: Solid line = General election; dashed line = presidential election; thick line = election and turnover of power.

B: Survey sample

The sampling frame was built on a previous face-to-face survey. as well as two phone surveys fielded since 2019 (Lust et al., 2021). The original survey sample was obtained via a stratified multistage probability proportional to size sampling scheme. The strata included two regions: 1) a 50km radius of Lusaka. and 2) a 100km region from the Zambia-Malawi border (Figure B1). Our survey sample is heavily. but not entirely. concentrated in the originally surveys locations (Figure B2). In the case that respondents moved out of the originally sampled areas. they may still appear in our survey sample. ...

Table B1 below summarizes the response rates for each round of the survey (ZEPS). For Round 1. we report two numbers for the size of the sampling frame. The first number is the total number of possible telephone numbers. The second number in parentheses is the number of telephone numbers associated with individuals who have taken at least one of the telephone surveys in addition to the face-to-face survey or were added as a new respondent during one of the telephone surveys. After the first round . new respondents were no longer accepted. therefore the size of the sampling frame decreases for rounds 2 and 3.

Although our sample is not nationally representative. the comparison of demographic variables with an Afrobarometer survey conducted approximately eight months prior to our survey illustrates that it is similar on dimensions such as gender and education. but less so in terms of age and location (urban/rural) (Table B2). For additional context. we also include a question on respondents' intended vote choice. However. here the comparability is limited given the timing of the surveys and the campaign season starting in May 2021 (Afrobarometer: November/December

2020 and ZEPS: July/August 2021). Nevertheless. the support for the UPND is remarkably similar in both surveys.

Table B1. Survey sample | respondents per round

	Round 1	Round 2	Round 3
Size of Sampling Frame	4226<br>(2603)	1674	1493
Sample Size	1692	1536	1299
Response Rate	40%<br>(65%)	92%	87%

Source: Lust et al. 2021

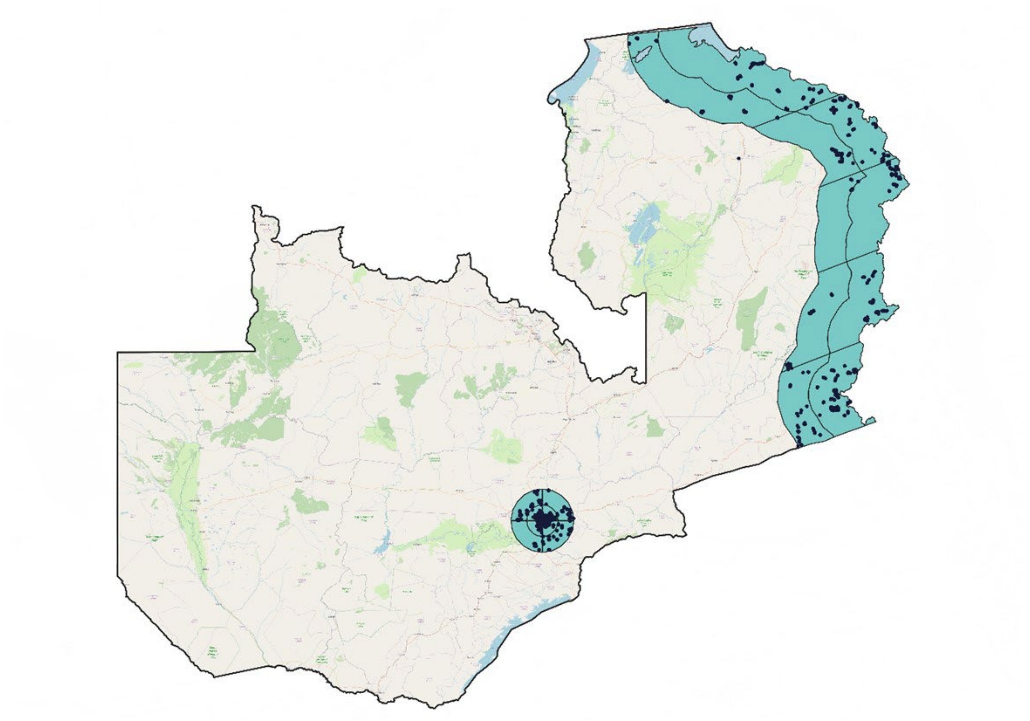

Figure B1. Sample map of original face-to-face survey. Source: Lust et al. 2021.

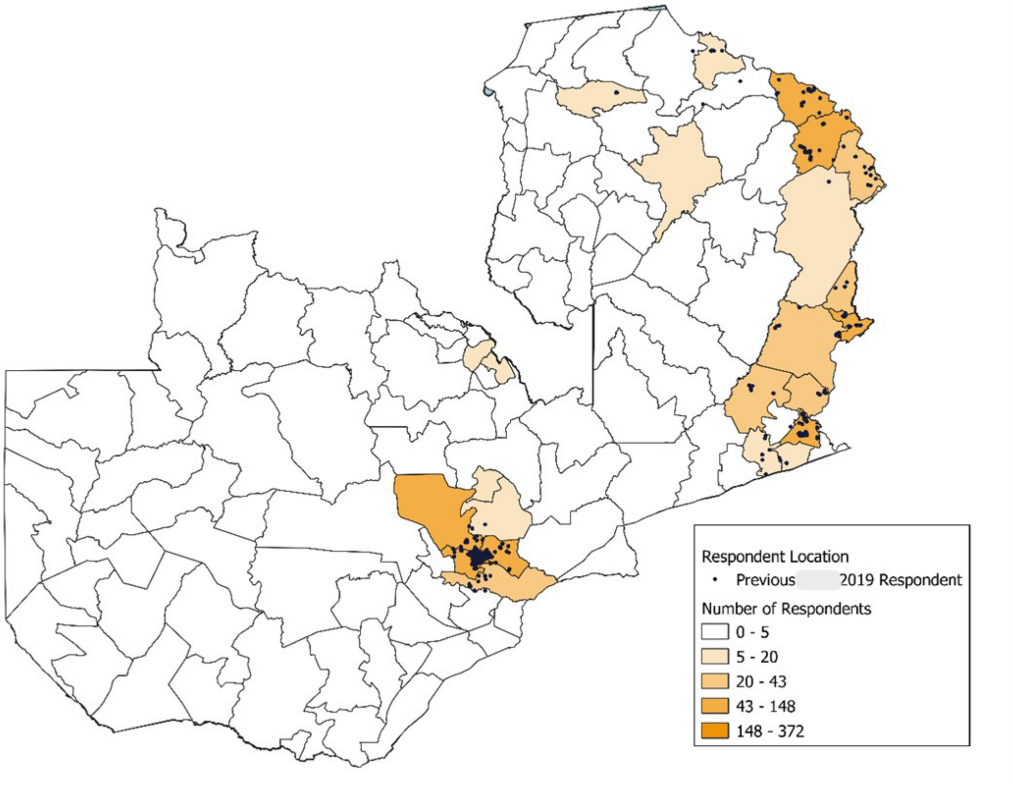

Figure B2. Round 3 heat map with an overlay of the locations of respondents who took the face-to-face survey. Source: Lust et al. 2021.

Table B2. ZEPS and Afrobarometer sample comparison

	**Zambian Election Panel Survey**	**Afrobarometer**	**Percentage point difference**
Gender	Female: 46%<br>Male: 54%	Female: 50%<br>Male: 50%	Female: -4<br>Male: +4
Age	18-24: 17%<br>25-34: 35%<br>35-44: 24%<br>45-54: 13%<br>55-64: 7%<br>65+: 3%	18-24: 26%<br>25-34: 29%<br>35-44: 21%<br>45-54: 13%<br>55-64: 7%<br>65+: 4%	18-24: -12<br>25-34: +7<br>35-44: +3<br>45-54: +1<br>55-64: 0<br>65+: 0
Location	Urban: 32%<br>Peri-urban: 30%<br>Rural: 39%	Urban: 45%<br>Peri-urban: NA<br>Rural: 55%	Urban: -13<br>Peri-urban: NA<br>Rural: -16
Education	No formal: 2%<br>Primary: 28%<br>Secondary: 53%<br>Post-secondary: 17%	No formal: 5%<br>Primary: 33%<br>Secondary: 45%<br>Post-secondary: 17%	No formal: -3<br>Primary: -5<br>Secondary: +8<br>Post-secondary: 0
Intended vote choice*	PF: 35%<br>UPND: 27%<br>NA/Refused/DK: 37%	PF: 23%<br>UPND: 25%<br>NA/Refused/DK: 51%	PF: +12<br>UPND: +2<br>NA/Refused/DK: -14

Source: Lust et al. 2032. ZEPS (July/August 2021). Afrobarometer Round 8 (November/December 2020).

Note: Percentage point differences might not balance out due to rounding of numbers. *The results for this question are only partially comparable as the Afrobarometer survey was fielded before the election campaign started. while the results from the ZEPS survey are drawn from Wave 2. which took place during the campaign season. In both surveys. respondents were asked: “If the presidential elections were held tomorrow. which party’s candidate would you vote for?”

C: Descriptive statistics and robustness checks

Table C1. Variable names and coding.

Variables	Question Wording	Variable Coding
Change election quality (R3-R2)	(R2) On the whole. how free and fair do you expect that the August 2021 presidential election will be?<br><br>(R3) On the whole. how free and fair do you think that the August 2021 presidential election was?	R2 and R3 indicators recoded: 0 "Not free and fair" 1 "Free and fair with major problems" 2 "Free and fair. but with minor problems" 3 "Completely free and fair." To create change in election quality. subtract R2 indicator from R3.
Vote Intentions (R2)	If the presidential elections were held tomorrow. which party's candidate would you vote for?	0 "UPND". 1 "PF". 2 "Abstain" Missing (Other Parties. DK/Refused)
Strength of Party Affiliation (R2)	I would like to know what you [factor] think of some different parties. How much do you like or dislike the United Party for National Development (UPND/PF) on a scale between 0 and 10. where 0 means Strongly dislike and 10 means strongly like.	Subtract the PF indicator from the UPND indicator. Then code strength of party affiliation: -10 to -4 (Strong PF supporters) -3 to -1 (Weak PF supporters); 10 to 4 (Strong UPND supporters); 3-1 (Weak PF supporters); 0= neutrals; missing values: Missing
Age (R1)	Respondent Age	
Female (R1)	Respondent Gender	Recoded: 0 "Male" 1 "Female"
Education (R1)	What is your highest level of education?	Recoded 1-10: 10 indicating "Post-graduate"
Urban/Peri-Urban Resident (R1)	Is your current village/neighborhood urban. peri-urban. or rural?	Recoded: 0 "Rural" 1 "Urban/Peri-Urban
Income (low)	I will read out a few statements about your income. Please tell me. which of the following statement is closest to your situation TODAY: 1) Our household income covers the needs well - we can save. 2) Our household income covers the needs alright. without much difficulty.3) Our household income does not cover the needs. there are difficulties.<br><br>4) Our household income does not cover the needs. there are great difficulties. 5) Don't Know/Refuse to Answer	Recoded: 0-4: 4 indicating "Our household income does not cover the needs. there are great difficulties"

EC Procedural Capacity Index (6items) (R3)	Level of satisfaction with ECZ's management of election processes at their polling station. including 1) accuracy of new voter register; 2) privacy of voting booths; 3) competence of polling staff; 4) impartiality of polling staff; 5) length of time it took to vote; 6) transparency of the counting and announcement of results.	Additive index rescaled 0-1: 1 indicating high levels of satisfaction.
Fear Violence (R2)	During the upcoming election. how much do you personally fear becoming a victim of political intimidation or violence?	Recoded 0-3: 3 indicating fear violence "A lot"
Know MP (R1)	Can you tell me the name of your Member of Parliament?	Recoded 0-1: 1 Correct name of MP; 0 Incorrect name/ could not name
Poor Government economic Performance (R2)	Since 2016 how well or badly would you say that President Lungu has managed the economy?	Recoded 0-1: 0 indicating well; 1 indicating badly

Table C2.1: Descriptive statistics ZEPS sample.

Variables	-1<br>N	-2<br>mean	-3<br>sd	-4<br>min	-5<br>max
Election quality (R1)	1573	1.951	1.034	0	3
Election quality (R2)	1452	2.011	0.944	0	3
Election quality (R3)	1529	2.498	0.705	0	3
Change election quality (R3-R2)	1209	0.565	1.093	-3	3
Age (R1)	1537	37.40	12.72	18	92
Female (R1)	1537	0.460	0.499	0	1
Education (R1)	1774	5.114	1.540	1	10
Urban/Peri-Urban Resident (R1)	1783	0.616	0.487	0	1
Know MP (R1)	1508	0.684	0.465	0	1
Income (low) (R1)	1760	2.143	0.809	0	3
Vote Intentions (R2)	1519	1.107	0.797	0	2
Party Affiliation (R2)	1376	2.023	1.494	0	4
Fear Violence (R2)	1535	1.334	1.241	0	3
EC Procedural Capacity Index (6-items) (R3)	1359	0.870	0.168	0	1
Economic performance	1494	0.569	0.495	0	1

Table C2.2 Descriptive Statistics ZEPS sample. Vote intention and party affiliation

	N	Response
Vote intention (R2)	1537	PF: 35%<br>UPND: 27%<br>Other: 1%<br>Abstain (NA/Refused/DK): 37%
Party affiliation (R2)	1279	Strong PF: 521 (41%)<br>Weak PF: 193 (15%)<br>Neutral: 284 (22%)<br>Weak UPND: 170 (13%)<br>Strong UPND: 395 (31%)<br>[Missing 234]

Table C3. Perceptions of Election Quality (Round 2 by Round 3). Cell entries are counts, row percentages, and column percentages.

	Round 3				
**Round 2**	Not free and fair	Free and fair with major problems	Free and fair with minor problems	Completely free and fair	**Total**
Not free and fair	10	4	19	44	**77**
	12.99	5.19	24.68	57.14	**100**
	23.81	7.41	7.2	5.18	**6.37**
Free and fair with major problems	9	24	65	182	**280**
	3.21	8.57	23.21	65	**100**
	21.43	44.44	24.62	21.44	**23.16**
Free and fair with minor problems	8	11	119	252	**390**
	2.05	2.82	30.51	64.62	**100**
	19.05	20.37	45.08	29.68	**32.26**
Completely free and fair	15	15	61	371	**462**
	3.25	3.25	13.2	80.3	**100**
	35.71	27.78	23.11	43.7	**38.21**
**Total**	**42**	**54**	**264**	**849**	**1.209**
	**3.47**	**4.47**	**21.84**	**70.22**	**100**
	**100**	**100**	**100**	**100**	**100**

Table C4. Regression analysis | change in perceptions of election quality (Ordered Logit)

	(1)	(2)
	Model_RB1	
	Change in perceived electoral quality of presidential election R3-R2	
VARIABLES	coef	pval
Vote Choice Pres (R2) = 0, UPND	0.955	(0.000)
Vote Choice Pres (R2) = 2, Abstain	0.418	(0.003)
ECZ Procedural Capacity	1.018	(0.003)
Fear Violence (R2)	0.257	(0.000)
Know MP (R1)	-0.299	(0.023)
Age (R1)	0.007	(0.140)
Female (R1)	-0.037	(0.764)
Education (R1)	0.026	(0.568)
Urban/Peri-Urban Resident (R1)	0.051	(0.692)
Income (low)	0.127	(0.094)
Constant cut1	-2.256	(0.000)
Constant cut2	-1.404	(0.010)
Constant cut3	-0.252	(0.634)
Constant cut4	2.390	(0.000)
Constant cut5	3.807	(0.000)
Constant cut6	5.753	(0.000)
Observations	1,015	

Note: Ordered Logistic regression.

Table C5: Regression analysis | change in perceptions of election quality (clustered standard errors: constituency).

	(1)	(2)
VARIABLES	Model_RB2<br>Change in perceived electoral quality of presidential election R3-R2	pval
Vote Choice Pres (R2) = 0, UPND	0.504	(0.000)
Vote Choice Pres (R2) = 2, Abstain	0.231	(0.005)
ECZ Procedural Capacity	0.545	(0.024)
Fear Violence (R2)	0.123	(0.000)
Know MP (R1)	-0.172	(0.051)
Age (R1)	0.003	(0.263)
Female (R1)	-0.041	(0.558)
Education (R1)	-0.001	(0.981)
Urban/Peri-Urban Resident (R1)	0.022	(0.749)
Income (low)	0.073	(0.079)
Constant	-0.491	(0.136)
Observations	953	
R-squared	0.112	

Note: OLS regression. Clustered by respondents’ constituency.

Table C6: Regression analysis | change in perceptions of election quality (R2 election quality measure included).

	(1)	(2)	(3)	(4)
	model_6a		model_6a	
VARIABLES	Change in perceived electoral quality of presidential election R3-R2	pval	Change in perceived electoral quality of presidential election R3-R2	pval
Perceptions of EQ (R2)	-0.877	(0.000)	-0.928	(0.000)
Vote Choice Pres (R2) = 0, UPND	0.048	(0.373)	0.065	(0.239)
Vote Choice Pres (R2) = 2, Abstain	0.077	(0.121)	0.090	(0.082)
ECZ Procedural Capacity			0.989	(0.000)
Fear Violence (R2)			-0.003	(0.769)
Know MP (R1)			-0.141	(0.004)
Age (R1)			0.000	(0.866)
Female (R1)			-0.021	(0.640)
Education (R1)			-0.041	(0.014)
Urban/Peri-Urban Resident (R1)			-0.029	(0.543)
Income (low)			0.004	(0.886)
Constant	2.301	(0.000)	1.872	(0.000)
Observations	1,200		1,015	
R-squared	0.563		0.616	

Note: OLS regression.

Table C7: Regression analysis | perceptions of election quality R3 (R2 election quality measure included)

VARIABLES	(1)<br>model_7a<br>Perceived electoral quality of presidential election R3	(2)<br>pval	(3)<br>model_7a<br>Perceived electoral quality of presidential election R3	(4)<br>pval
Perceptions of EQ (R2)	0.123	(0.000)	0.072	(0.005)
Vote Choice Pres (R2) = 0, UPND	0.048	(0.373)	0.065	(0.239)
Vote Choice Pres (R2) = 2, Abstain	0.077	(0.121)	0.090	(0.082)
ECZ Procedural Capacity			0.989	(0.000)
Fear Violence (R2)			-0.003	(0.769)
Know MP (R1)			-0.141	(0.004)
Age (R1)			0.000	(0.866)
Female (R1)			-0.021	(0.640)
Education (R1)			-0.041	(0.014)
Urban/Peri-Urban Resident (R1)			-0.029	(0.543)
Income (low)			0.004	(0.886)
Constant	2.301	(0.000)	1.872	(0.000)
Observations	1,200		1,015	
R-squared	0.025		0.115	

Table C8: Regression analysis | change in perceptions of election quality (strength of party affiliation).

	(1)	(2)
	Model_RB1	
	Change in perceived electoral quality of presidential election R3-R2	
VARIABLES	coef	pval
Vote Choice Pres (R2) = 0, UPND	0.955	(0.000)
Vote Choice Pres (R2) = 2, Abstain	0.418	(0.003)
ECZ Procedural Capacity	1.018	(0.003)
Fear Violence (R2)	0.257	(0.000)
Know MP (R1)	-0.299	(0.023)
Age (R1)	0.007	(0.140)
Female (R1)	-0.037	(0.764)
Education (R1)	0.026	(0.568)
Urban/Peri-Urban Resident (R1)	0.051	(0.692)
Income (low)	0.127	(0.094)
Constant cut1	-2.256	(0.000)
Constant cut2	-1.404	(0.010)
Constant cut3	-0.252	(0.634)
Constant cut4	2.390	(0.000)
Constant cut5	3.807	(0.000)
Constant cut6	5.753	(0.000)
Observations	1,015	

Note: OLS regression.

Table C9: Regression analysis | change in perceptions of election quality (R2 government economic performance included).

	(1)	(2)
	Model_10a	
VARIABLES	Change in perceived electoral quality of presidential election R3-R2	pval
Vote Choice Pres (R2) = 0, UPND	0.355	(0.000)
Vote Choice Pres (R2) = 2, Abstain	0.162	(0.044)
ECZ Procedural Capacity	0.679	(0.000)
Fear Violence (R2)	0.123	(0.000)
Know MP (R1)	-0.145	(0.048)
Age (R1)	0.004	(0.191)
Female (R1)	-0.047	(0.495)
Education (R1)	-0.000	(0.993)
Urban/Peri-Urban Resident (R1)	0.056	(0.435)
Income (low)	0.051	(0.217)
RECODE of q5_R2	0.305	(0.000)
Constant	-0.725	(0.012)
Observations	990	
R-squared	0.137	

Note: OLS regression.

Table C10: Regression analysis | change in perceptions of election quality (R2 UPND Supporters with high perceived electoral quality removed).

	(1)	(2)
	Model_11a	
VARIABLES	Change in perceived electoral quality of presidential election R3-R2	pval
Vote Choice Pres (R2) = 0, UPND	0.662	(0.000)
Vote Choice Pres (R2) = 2, Abstain	0.238	(0.003)
ECZ Procedural Capacity	0.678	(0.001)
Fear Violence (R2)	0.121	(0.000)
Know MP (R1)	-0.210	(0.008)
Age (R1)	0.002	(0.409)
Female (R1)	-0.070	(0.350)
Education (R1)	-0.010	(0.718)
Urban/Peri-Urban Resident (R1)	0.050	(0.515)
Income (low)	0.058	(0.192)
Constant	-0.493	(0.107)
Observations	916	
R-squared	0.132	

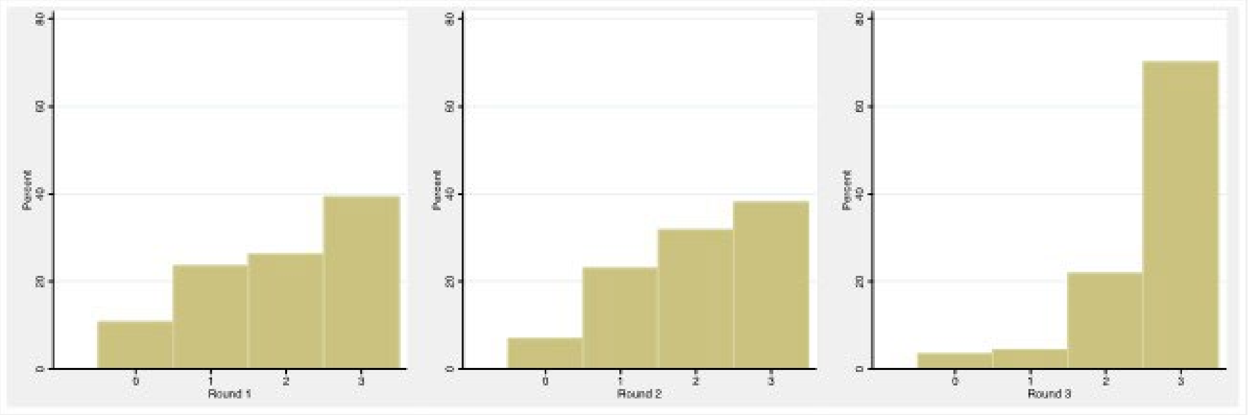

Figure C1. Histogram of perceptions of election quality | Round 1,2 & 3. Based on respondents who answered questions about perceptions of election quality in all three rounds. Responses options include: Not free and fair" 1 "Free and fair with major problems" 2 "Free and fair. but with minor problems" 3 "Completely free and fair."

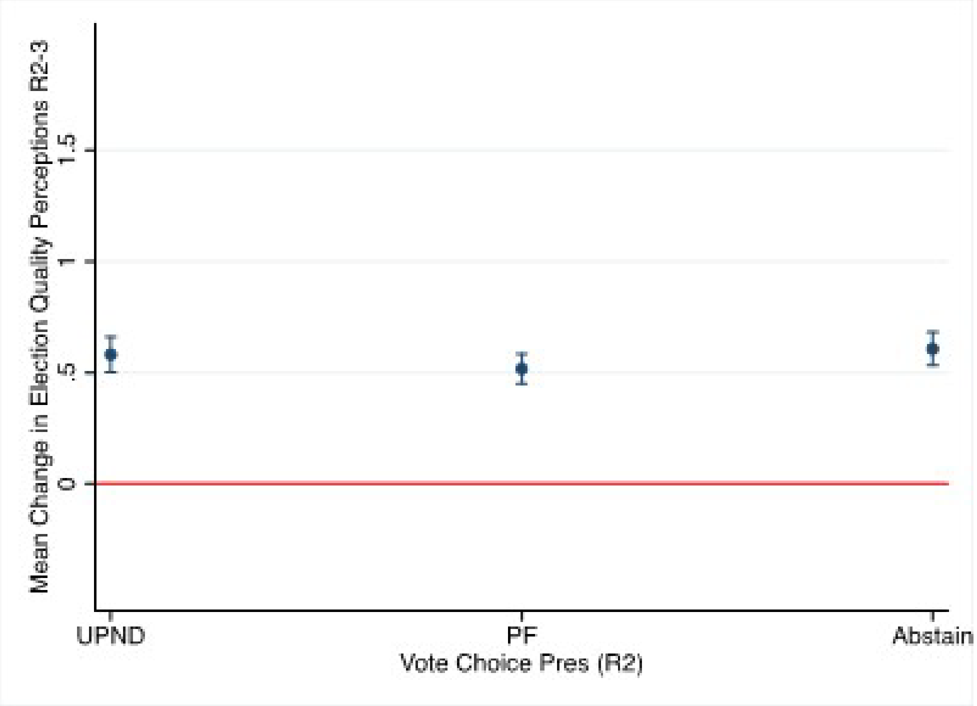

Figure C2. Predicted mean changes in election quality perceptions (R3-R2) by intended vote choice. R2 election quality measure included. Marginsplot illustrates how the predicted mean change in election quality perceptions (R2) varies by respondents' intended vote choice for the main political parties (based on Table C6 Model 2). Vertical bars indicate 95% confidence intervals.

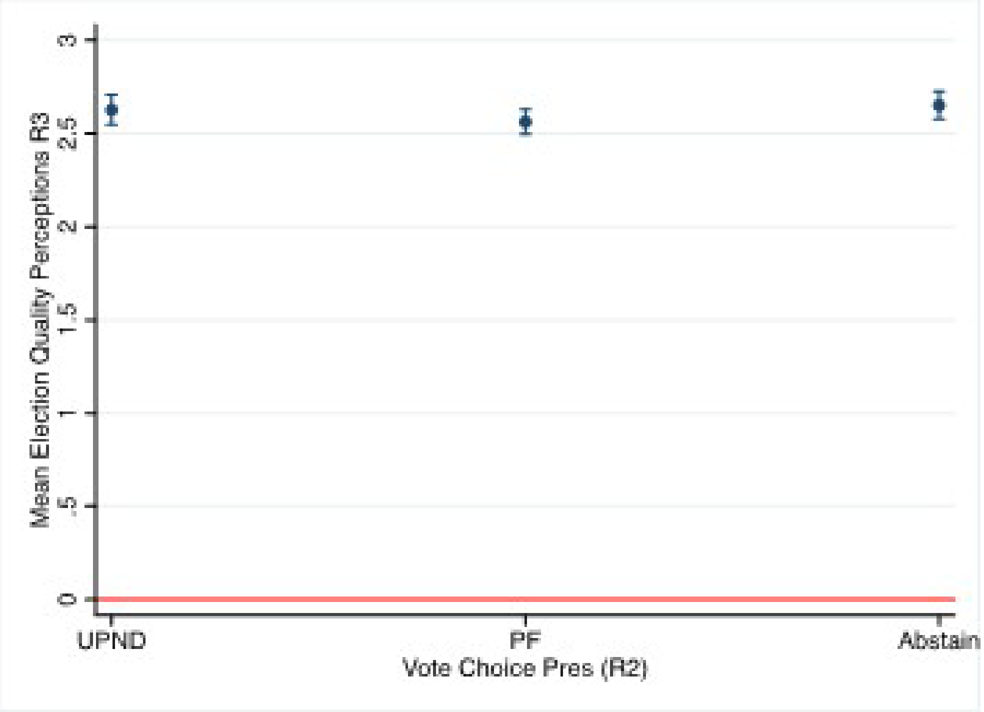

Figure C3: Predicted mean election quality perceptions (R3) by intended vote choice. R2 election quality measure included. Marginsplot illustrates how the predicted mean election quality perceptions (R3) varies by respondents' intended vote choice for the main political parties (based on Table C7 Model 2). Vertical bars indicate 95% confidence intervals.
